# 

**Published:** 1906-01-20

**Authors:** 


### NOTICE TO CORRESPONDENTS.

All MSS., Letters, Books for Review, and other matters intended for the Editor should be addressed The Editor, "The Hospital," The Hospital Buildings, 28 & 29 Southampton Street, Strand, W.C.

The Editor cannot undertake to return rejected MS., even when accompanied by stamped directed envelope.

**Notice to Advertisers and Subscribers.**

All Advertisements, Orders for Copies of The Hospital, and Business Communications should be addressed to **THE MANAGER (Not to the Editor),** The Hospital, 28 & 29 Southampton Street, Strand London, W.C.

The Cost of Subscription, post free, is as follows:—Per week, 3½d.; per quarter, 3s. 3d.; per half-year, 6s. 6d.; per annum, 13s. Foreign and Colonial:—Per annum, 19s. 6d., payable, in all cases, in advance.

**NOTICE.—Vol. XXXVIII. of "The Hospital" April to September 1905), in handsome cloth binding, will shortly be on sale at the office of this Journal, price 10s. 6d. Cases for binding the Numbers contained in this Volume 2s. Index to Vol. XXXVIII., 3½d., post free.**

**The Monthly Part for January is now ready. Price 1s., by post, 1s. 3d.**

### Medical Appointments and Vacancies.

### VACANCIES.

Bridge of Weir, Consumption Sanatoria and Colony of Mercy for Epileptics.—Medical Superintendent.
Bristol, City and County of.—Public Analyst.
Burnley, County Borough of.—Medical Officer of Health.
Cheltenham General Hospital.—Junior House Surgeon.
Chesterfield and North Derbyshire Hospital and Dispensary.—Junior House Surgeon.
Glasgow, Corporation of.—Lady Medical Assistant.
Great Northern Central Hospital, Holloway, N.—Two Anæsthetists.
Hospital for Consumption and Diseases of the Chest, Brompton.—Resident House Physicians.
King's College (University of London).—Sambrooke Surgical Registrar.
New Hospital for Women.—Senior Assistant to the Children's Out-patient Department. Also two Clinical Assistants.
Parish of St. Mary, Islington.—Medical Officer and Public Vaccinator for No. 9 District.
Seamen's Hospital Society.—Pathologist, Assistant Physician in charge of the Electrical Department, Medical Registrar, Surgical Registrar, and Honorary Anæsthetists.
University of London.—Examiners in Medicine, in Surgery, and in Pathology.
Westminster Hospital, Broad Sanctuary, S.W.—Fourth Assistant Surgeon.
York County Hospital.—House Surgeon.

SEAMEN'S HOSPITAL SOCIETY.

THE SIGN OF A GOOD NURSING MANUAL.

### NURSE.

DO YOU KNOW THAT YOU CAN OBTAIN

**NURSING MANUALS - - -**

**CASE-BOOKS and - - - -**

**CHARTS - - - - - - -**

OF ALL DESCRIPTIONS OF

***THE SCIENTIFIC PRESS, LTD.,***

**28 & 29 Southampton Street, Strand, London, W.C. ?**

***A Complete Guide to the Nursing Profession***

**HOW TO BECOME A NURSE: How and Where to Train.** By Sir Henry Burdett, K.C.B. 2/- net, 2/4 post free.

***The Duties of a Nurse Explained***

**NURSING HINTS TO PROBATIONERS ON PRACTICAL WORK.** By M. Annesley Voysey. 2/- net, 2/3 post free.

The *Lancet* says: "This is a good practical book. . . . It contains the whole duty of Nurses given in plain language."

***INDISPENSABLE HANDBOOKS FOR PROBATIONERS.***

***Bandaging***

**A PRACTICAL GUIDE TO SURGICAL BANDAGING AND DRESSINGS.** By Wm. Johnson Smith, F.R.C.S. Suitable size for the apron pocket. 2/- post free.

**SURGICAL INSTRUMENTS AND APPLIANCES USED IN OPERATIONS.** A Classified List, with Explanatory Notes. Illustrated. 1/6 net, 1/8 post free.

***Dictionary***

**THE NURSE'S PRONOUNCING DICTIONARY OF MEDICAL TERMS & NURSING TREATMENT.** Compiled by Honnor Morten. New Edition, edited by Mary I. Burdett. Suitable size for apron pocket. 2/- post free.

***Anatomy and Physiology***

**ELEMENTARY ANATOMY AND SURGERY FOR NURSES.** By William McAdam Eccles, M.S. Lond., M.B. Profusely Illustrated. 2/6 post free.

**ELEMENTARY PHYSIOLOGY FOR NURSES.** By C. F. Marshall, M.D., B.Sc., F.R.C.S. Illustrated. 2/- post free.

**ELEMENTS OF ANATOMY AND PHYSIOLOGY.** By W. Bernard Secretan, M.D. Profusely Illustrated. Cloth, 2/- net, 2/3 post free.

***Special Nursing***

**OPHTHALMIC NURSING.** By Sydney Stephenson, M.B., F.R.C.S. New and Revised Edition. 3/6 net, 3/10 post free.

**THE MODERN NURSING OF CONSUMPTION.** By Dr. Jane Walker. 1/- net, 1/2 post free.

**NURSING IN DISEASES OF THE THROAT, NOSE, AND EAR.** By P. Macleod Yearsley, F.R.C.S. Eng., M.R.C.S., L.R.C.P. Lond. Illustrated. 2/6 post free.

**MENTAL NURSING.** By William Harding, M.D. Ed., M.R.C.P. Lond. New and Popular Edition. 1/- post free.

**FEVERS AND INFECTIOUS DISEASES: The Nursing and Practical Treatment.** By Wm. Harding, M.D. Ed., M.R.C.P. Lond. 1/- post free.

**The Scientific Press, Ltd.**

*Send for Complete Catalogue.*

**Publishers of Nursing Text-Books, Charts, and Case-Books of all Descriptions.**

**28 & 29 SOUTHAMPTON STREET, STRAND, LONDON, W.C.**

